# High-dose Intensity-modulated proton therapy versus Standard-dose Intensity-modulated RadIation therapy for esophageal squamous cell carcinoma (HI-SIRI): study protocol for a randomized controlled clinical trial

**DOI:** 10.1186/s13063-022-06822-8

**Published:** 2022-10-22

**Authors:** Chawalit Lertbutsayanukul, Sarin Kitpanit, Danita Kannarunimit, Chakkapong Chakkabat, Sornjarod Oonsiri, Kullathorn Thephamongkhol, Putipun Puataweepong, Kanyarat Katanyoo, Jirasak Sukhaboon, Chokaew Tovanabut, Sirikanya Chongsathientham, Pornravee Treeratsapanich, Jirarat Soonthornrak, Anussara Prayongrat

**Affiliations:** 1 Division of Radiation Oncology, Department of Radiology, Faculty of Medicine, Chulalongkorn University, King Chulalongkorn Memorial Hospital, 1873 Rama IV Road, Pathumwan, Bangkok, 10330 Thailand; 2 Division of Radiation Oncology, Department of Radiology, Faculty of Medicine, Siriraj Hospital, Mahidol University, Bangkok, Thailand; 3 Division of Radiation Oncology, Department of Radiology, Ramathibodi Hospital, Mahidol University, Bangkok, Thailand; 4 Division of Radiation Oncology, Department of Radiology, Faculty of Medicine, Vajira Hospital, Navamindradhiraj University, Bangkok, Thailand; 5 Department of Therapeutic Radiology, Lopburi Cancer Hospital, Lopburi, Thailand; 6 Division of Radiation Oncology, Chonburi Cancer Hospital, Chonburi, Thailand; 7 Division of Radiation Oncology, National Cancer Institute, Bangkok, Thailand; 8 Division of Radiation Oncology, Chulaphorn Institute, Bangkok, Thailand; 9 Division of Radiation Oncology, Chanthaburi Cancer Hospital, Chantaburi, Thailand

**Keywords:** Intensity-modulated proton therapy, Intensity-modulated radiotherapy, Esophageal cancer, Squamous cell carcinoma, Survival, Toxicity

## Abstract

**Background:**

Chemoradiotherapy is the standard of care for esophageal cancer as a neoadjuvant treatment before surgery, or as a definitive treatment for unresectable disease. Intensity-modulated radiotherapy (IMRT) has been considered the standard radiation technique. However, patients suffer from treatment-related toxicities, and most die from disease progression or recurrence. With emerging technological advancement, proton therapy has theoretical advantages over IMRT because it offers apparent dosimetric benefits to allow dose escalation to the target while better sparing surrounding tissues such as the lungs, heart, liver, and spinal cord. The purpose of this study protocol is to investigate the survival benefit of proton therapy using modern intensity-modulated proton therapy (IMPT) compared to standard IMRT for esophageal cancer.

**Methods:**

This is a two-arm open phase II/III multi-institution randomized controlled trial. Eligible patients will have histologically confirmed squamous cell carcinoma of the thoracic esophagus with no evidence of tracheoesophageal/esophagobronchial fistula or distant metastasis. After stratification according to resectability status (resectable vs. borderline resectable/unresectable), a total of 232 patients will be randomized to receive IMPT or IMRT using a 1:1 allocation ratio. In resectable cases, surgical resection following concurrent chemoradiation will be attempted for the patients who are medically fit at the time of surgery. In those with initially borderline resectable/unresectable disease, definitive concurrent chemoradiation will be performed. The phase II study will assess safety (toxicity and postoperative complications) and feasibility (recruitment rate and chemoradiation dose modification) in 40 patients into each arm. The study will then continue into phase III, further recruit 76 patients into each arm, and compare progression-free survival between IMPT vs IMRT groups. The secondary endpoints will be overall survival, local and distant control, toxicities, health-related quality of life, and cost-utility. This protocol describes a detailed radiotherapy and chemotherapy.

**Discussion:**

This randomized clinical trial will demonstrate the clinical benefit of IMPT in esophageal cancer treatment in terms of survival and toxicity outcomes which will further establish high-level evidence for radiation modality in squamous cell carcinoma of the thoracic esophagus.

**Trial registration:**

TCTR20200310006. Registered 10 March 2020.

**Supplementary Information:**

The online version contains supplementary material available at 10.1186/s13063-022-06822-8.

## Administrative information

Note: the numbers in curly brackets in this protocol refer to SPIRIT checklist item numbers. The order of the items has been modified to group similar items (see http://www.equator-network.org/reporting-guidelines/spirit-2013-statement-defining-standard-protocol-items-for-clinical-trials/).

**Table:** 

Title {1}	Intensity-modulated radiotherapy (IMRT) vs intensity-modulated proton therapy (IMPT) for esophageal squamous cell carcinoma: study protocol for a randomized controlled clinical trial
Trial registration {2a and 2b}.	TCTR20200310006. Registered 10 March 2020
Protocol version {3}	1 August 2021, version 1
Funding {4}	This Research is funded by Thailand Science research and Innovation Fund Chulalongkorn University CU_FRB65_hea (30)_037_30_18
Author details {5a}	**Authors** Chawalit Lertbutsayanukul^1^, Sarin Kitpanit^1^, Danita Kannarunimit^1^, Chakkapong Chakkabat^1^, Sornjarod Oonsiri^1^, Kullathorn Thephamongkhol^2^, Putipun Puataweepong^3^, Kanyarat Katanyoo^4^, Jirasak Sukhaboon^5^, Chokaew Tovanabut^6^, Sirikanya Chongsathientham^7^, Pornravee Treeratsapanich^8^, Jirarat Soonthornrak^9^, Anussara Prayongrat^1^* **Affiliations** ^1^Division of Radiation Oncology, Department of Radiology, Faculty of Medicine, Chulalongkorn University, King Chulalongkorn Memorial Hospital, Bangkok, Thailand, ^2^Division of Radiation Oncology, Department of Radiology, Faculty of Medicine, Siriraj Hospital, Mahidol University, Bangkok, Thailand, ^3^Division of Radiation Oncology, Department of Radiology, Ramathibodi Hospital, Mahidol University, Bangkok, Thailand, ^4^Division of Radiation Oncology, Department of Radiology, Faculty of Medicine, Vajira Hospital, Navamindradhiraj University, Bangkok, Thailand, ^5^Department of Therapeutic Radiology, Lopburi Cancer Hospital, Lopburi, Thailand, ^6^Division of Radiation Oncology, Chonburi Cancer Hospital, Chonburi, Thailand, ^7^Division of Radiation Oncology, National Cancer Institute, Bangkok, Thailand, ^8^Division of Radiation Oncology, Chulaphorn Institute, Bangkok, Thailand, ^9^Division of Radiation Oncology, Chantaburi Cancer Hospital, Chantaburi, Thailand
Name and contact information for the trial sponsor {5b}	None
Role of sponsor/funder {5c}	The funder played no part in study design; collection, management, analysis, and interpretation of data; writing of the report; and the decision to submit the report for publication

## Introduction

### Background and rationale {6a}

Esophageal cancer is the sixth leading cause of death and the eighth most common cancer worldwide [1]. Esophagectomy is a mainstay of curative treatment, but only 30–40% of patients have potentially resectable disease and require neoadjuvant chemoradiotherapy (CRT). The remainders are unresectable and need definitive CRT, but the locoregional failure rate is more than 50% in locally advanced diseases [2, 3]. To overcome recurrence, two options including salvage esophagectomy, so-called trimodality treatment [4–6], and high-dose CRT [7–9] are suggested but come with the cost of treatment-related complications. Our experience using neoadjuvant high-dose (≥60Gy) CRT followed by esophagectomy in thoracic esophageal squamous cell carcinoma (ESCC) revealed a high pathologic complete remission rate (59%) with favorable survival, compared with <60Gy [10]. However, a recent randomized controlled trial (RCT), ARTDECO study, revealed no survival benefit from high-dose intensity-modulated radiotherapy (IMRT) over standard doses (61.6 Gy vs 50.4 Gy) which might be due to a slightly higher grade 5 toxicity high-dose arm (8% vs 3%) [11].

Technological advancements in radiotherapy (RT) have been improving to deliver high radiation doses to tumors while minimizing doses to normal structures such as the lungs, heart, and spinal cord. In comparison with IMRT, proton beam therapy (PBT) has profound dosimetric advantages that allow dose escalation to the target and improving disease control while minimizing radiation-induced complications [12–16]. Two retrospective studies revealed that PBT had better overall survival (OS) and lower doses to the heart and lungs than IMRT, which translated into a lower incidence of grade ≥3 cardiac and pulmonary toxicities [17, 18]. Moreover, reduction of integral dose might reduce the risk of lymphopenia, leading to improved immune surveillance and, possibly, survival [19–21]. Recently, the first RCT comparing between PBT and IMRT from MD Anderson Cancer Center demonstrated similar 3-year progression-free survival (PFS) and OS with a significantly lower toxicity in the PBT group [22]. This trial was early terminated due to high drop-out rate but anticipate the opening of NRG-GI006 in March 2019 (Phase III Randomized Trial of Proton Beam Therapy Versus Intensity Modulated Photon Radiotherapy for the Treatment of Esophageal Cancer, NCT03801876).

However, majority of patients in Western countries had adenocarcinoma of distal esophageal cancer or gastroesophageal junction tumors. In contrast, ESCC is a more common histology in the Asian population [1] with evidence of a dose-response relationship [10, 23] and predominantly locates in the upper to mid-thoracic region where surgery is less feasible. Hence, a dose escalation regimen is potentially beneficial for these patients and has shown to be administered safely by PBT in Asian studies [24–26]. Furthermore, previous PBT studies reported outcomes based on a conventional technique, passive scattering proton therapy, which delivered less dose conformity than a modern pencil-beam scanning or intensity-modulated proton therapy (IMPT). But there are many challenges for IMPT in esophageal cancer treatment, e.g., heterogeneous density at the tumor-lung interface, interplay effects due to respiration and cardiac motion, and anatomical changes during the treatment course [27, 28]. Thus far, only a few publications reported the clinical outcomes of this technique in esophageal cancer [29, 30]. Therefore, we aim to investigate the clinical benefit of IMPT in comparison with IMRT, for the treatment of thoracic ESCC.

### Objectives {7}

The primary endpoint is to compare the progression-free survival (PFS) between IMPT versus IMRT for the treatment of thoracic ESCC. The hypothesis of this study is that high-dose IMPT (≥60Gy) is feasible, safe, and potentially improved PFS by 20% compared with standard-dose IMRT. The secondary endpoints include OS, locoregional failure-free survival (LRFFS), distant metastasis-free survival (DMFS), and treatment-related toxicities. Other prespecified endpoints are surgical outcomes (pathological tumor response, hospital stay, perioperative complications, and mortality) for resectable cases, health-related quality of life, and cost-utility analysis. The final goal of this RCT is to establish high-level evidence for the implication of IMPT for ESCC treatment.

### Trial design {8}

This HI-SIRI trial is a multi-institutional, randomized, phase II/III superiority trial. The calculated sample size will be 232 patients (116 in each group) to detect a 20% increase in progression-free survival (PFS) with *α* = 0.05 and power = 80%, corrected for a 10% dropout rate. The patient will be stratified according to resectability status (resectable vs. borderline resectable/unresectable) evaluated by experienced surgeons and, subsequently, will be randomized to receive IMPT or IMRT using a 1:1 allocation ratio. In resectable cases, neoadjuvant CRT followed by surgery will be attempted in the patients who are medically fit at the time of surgery. In those with unresectable disease, definitive CRT will be performed. The phase II study will focus on the efficacy and safety of IMPT with chemotherapy in 40 patients in each arm as well as the feasibility of the study (recruitment rate and CRT dose modification). Thereafter, further patient accrual of 76 patients for each arm will continue with a 2-year follow-up.

## Methods: participants, interventions, and outcomes

### Study setting {9}

To establish the national guideline of PBT in Thailand, a multi-institutional study will be performed in 9 institutions, both academic and community-based hospitals, throughout Thailand (Bangkok, Chonburi, Lopburi, Chanthaburi province).

### Eligibility criteria {10}

The inclusion criteria are (i) biopsy-proven stage II–IVA squamous thoracic esophageal cancer, (ii) age 20–70, (iii) Eastern Cooperative Oncology Group (ECOG) 0–2, (iv) body weight > = 45kg at the time of study entry, and (v) normal blood, liver, and renal function. Patients are excluded if they have (i) distant metastasis, (ii) evidence of tracheoesophageal (TE) or esophagobronchial fistula (T4b), (iii) prior or simultaneous malignancies within the past 2 years (other than skin cancers), (iv) prior radiation treatment to the chest or mediastinum with overlapping fields, (v) uncontrolled intercurrent illness, and (vi) are pregnant or nursing.

### Who will take informed consent? {26a}

All patients who meet eligibility criteria will be registered with an individual code. The consent form, patient information sheet, and other related documents will be given to participants and authorized surrogates. Once informed consent is obtained by investigators, baseline data and study measurements will be collected. The participants will be stratified according to their resectability status and randomized into two treatment arms, IMPT versus IMRT, using the stratified block randomization method. Anonymity will be assured using covered envelopes.

### Additional consent provisions for collection and use of participant data and biological specimens {26b}

Not applicable. This study will not collect any participant biological specimens.

## Interventions

### Explanation for the choice of comparators {6b}

Arm A (experimental arm) is IMPT 50 Gy relative biological effectiveness (RBE) in 25 fractions followed by a shrinking field boost of 10–14 Gy in 5–7 fractions to the high-risk volume. The radiation will be given 2 Gy daily fraction for 5 fractions in 1 week.

Arm B (controlled arm) is IMRT 50–50.4 Gy in 25–28 fractions, given 1.8–2 Gy daily fraction for 5 fractions in 1 week. The simultaneous integrated boost (SIB) technique is allowed to give higher radiation (up to 54 Gy) to high-risk volume.

### Intervention description {11a}

#### Patient assessment schedule

All patients will undergo pretreatment evaluation consisting of physical examinations, nutritional assessment, esophagogastroduodenoscopy (EGD) with biopsy, and laboratory investigations (complete blood count, liver, and renal function). Endoscopic ultrasonography (EUS) with fine needle aspiration (FNA) of a suspicious lymph node is allowed. If the tumor is located above the carina with a suspected TE fistula, bronchoscopy will be required. Diagnostic radiology includes computer tomography (CT) of the chest and upper abdomen and bone scintigraphy, all of which can be substituted by positron emission tomography (PET)/CT scans. Furthermore, lung function tests will be indicated for patients with planned esophagectomy.

During treatment, toxicities will be assessed weekly during CRT and prior to each adjuvant chemotherapy cycle, using Common Terminology Criteria for Adverse Events (CTCAE) version 5. Appropriate management of toxicities will be provided accordingly. After treatment completion, the participants will be scheduled for clinical response, toxicity, and quality of life evaluation for every 3 months × 3 visits, and every 6 months × 2 visits, then annually. Diagnostic imaging and EGD with biopsy warranting consideration of residual, recurrent tumor, and metastatic disease will be conducted 12 weeks after radiation treatment completion. Primary tumor and nodal response will be evaluated by CT scan in all patients using Response Evaluation Criteria in Solid Tumours (RECIST) criteria version 1.1.

Details are described in Table 1.

**Table 1:** Patient assessment schedule

Diagnostic study	Pre-study^**a**^	Prior to CCRT	Weekly during CCRT	Prior to each CMT cycle	12 weeks after CCRT	Follow-up visits^**g**^
History and physical exam	x	x	x	x	x	x
Nutritional evaluation	x	x	x	x	x	x
Complete blood count	x^b^	x	x	x	x	x
Liver and renal function	x^b^	x		x		
Tumor markers	x					x
Bronchoscopy^c^	x					
EGD with biopsy	x				x^f^	x^f^
CT chest	x^d^				x	x
PET/CT scan^e^	x				x	x
Endoscopic ultrasound^e^	x				x^f^	x^f^
Bone scan^e^	x					x
Toxicity evaluation			x	x	x	x
QOL questionnaires			x		x	x
Pathological assessment, if surgery					x	

*CCRT* concurrent chemoradiation, *CMT* chemotherapy, *EGD* esophagogastroduodenoscope, *CT* computed tomography, *PET* positron emission tomography, *QOL* quality of life

^a^Pre-study should be completed within 6 weeks prior to study entry (radiation start)

^b^Should be taken within 14 days prior to study entry

^c^Required if the tumor is above the carina with suspected tracheoesophageal fistula

^d^CT ≤ 6 weeks prior to study entry. Treatment planning CT scan ≤ 3 weeks prior to radiation

^e^PET/CT scan and endoscopic ultrasound are optional. Bone scan is considered in patients who cannot perform PET/CT scan to exclude bone metastasis

^f^If clinically indicated

^g^Every 3 months × 3 visits, then every 6 months × 2 visits, then yearly

#### Treatment 

##### Radiation therapy

**Fig. 1:**
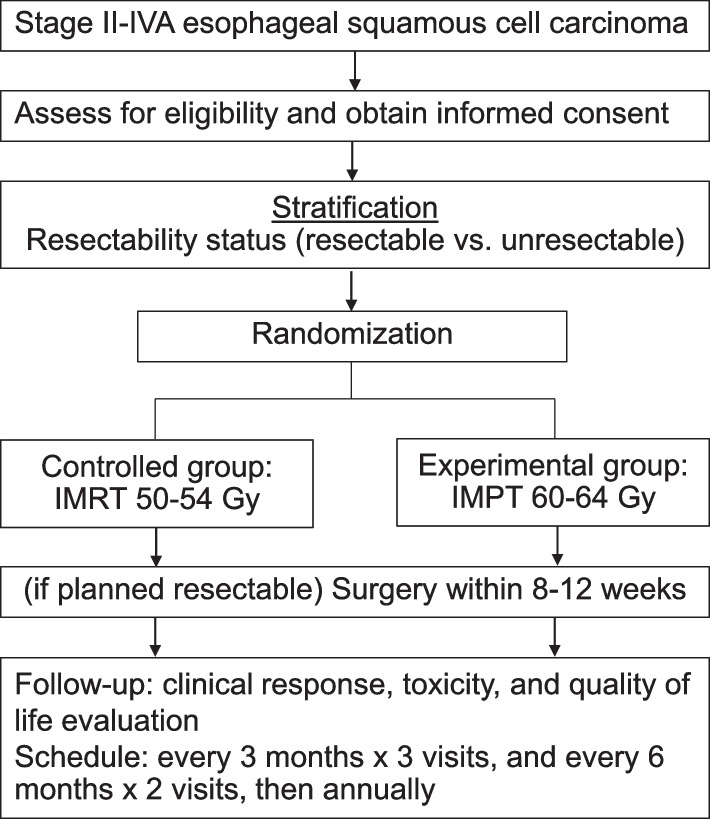
Schema of the clinical trial and study flow chart

**Fig. 2:**
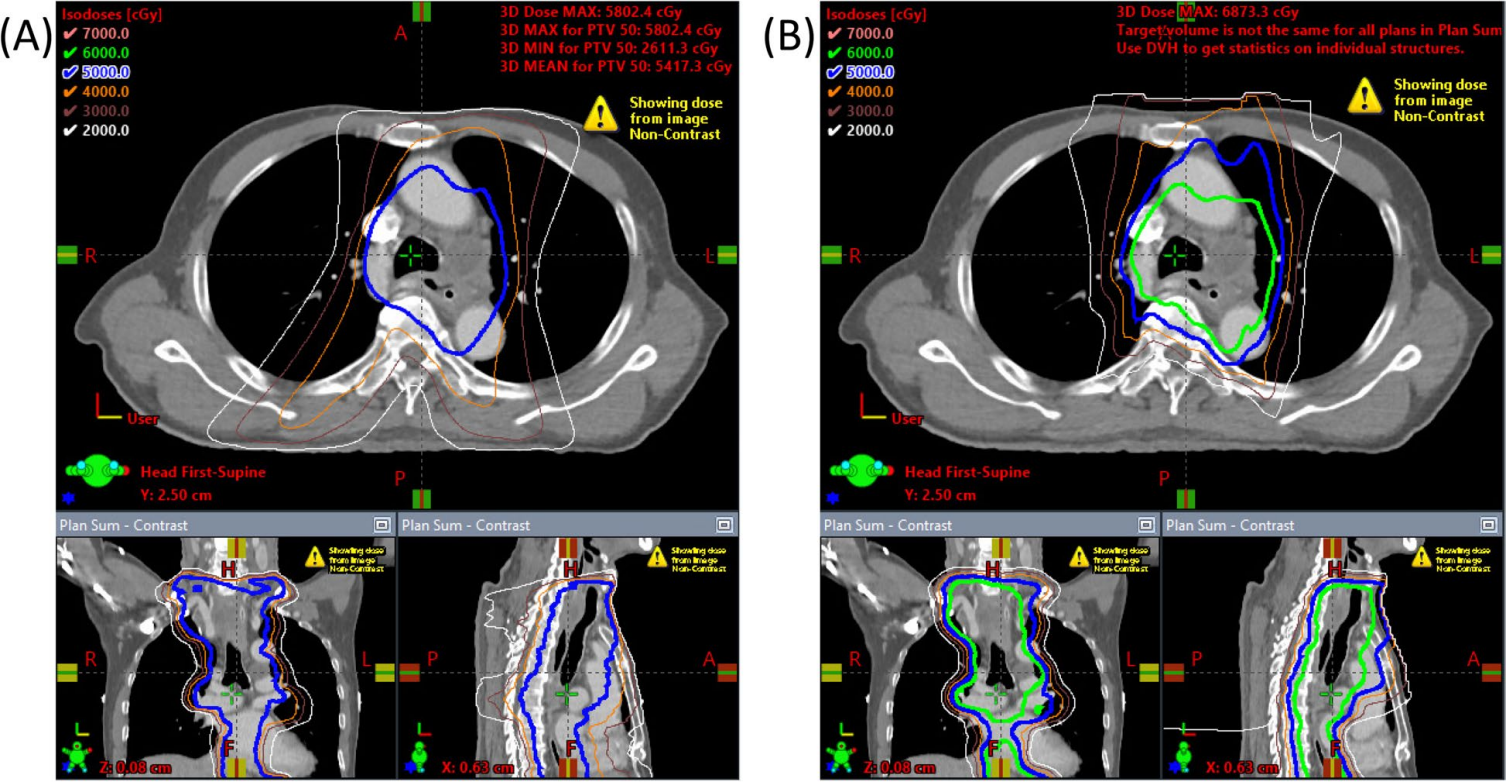
Example of IMRT versus IMPT plan of the mid thoracic esophageal cancer T3N2M0. (**A**) IMRT plan 50 Gy in 25 fractions (blue isodose line) and (**B**) IMPT plan 60 Gy in 30 fractions (green isodose line)

The patients are immobilized in the supine position using an extended wing board with their arms above their heads. When the supraclavicular lymph node is included in the target volume, head-shoulder thermoplastic masks will be applied with their arms straight beside their body. A four-dimensional (4D) CT simulation will be used to account for respiratory motion with a 2.5-mm slice thickness. The planning 4D CT images will be transferred to the treatment planning system and used and incorporated with EGD results and (PET/) CT scan for target delineation, if available. Target volumes, elective nodal level, normal structure delineation, and constraints are described in Supplement 1. An example of IMRT and IMPT plans is shown in Figs. 1 and 2.

##### Chemotherapy

Patients will undergo CRT with either of the following regimen: (i) cisplatin (80 mg/m^2^) and 5-fluorouracil (1000 mg/m^2^/day for 4 days) administered every 4 weeks for 2 cycles or (ii) carboplatin (area under curve 2 mg/ml/min) and paclitaxel (50 mg/m^2^) administered every 1 week for 5–6 cycles. For definitive CRT, adjuvant chemotherapy consisting of cisplatin and 5-fluorouracil will be administered at 4-week intervals for 2 cycles. For postoperative patients with residual pathologic disease, adjuvant nivolumab is allowed [31].

##### Esophagectomy

According to institutional protocol, surgery will be conducted within 8–12 weeks following the completion of neoadjuvant CRT. The type of surgery will depend on the location and extent of the primary tumor. Patients will undergo an esophagectomy with anastomotic reconstruction with complete intrathoracic nodal staging and a feeding tube for postoperative nutritional support.

If the cancer is unresectable or the patients are medically unfit at the time of surgery, the patients will be followed for tumor response, locoregional and distant control, and survival. Additional therapy will be based on the judgment of the treating physicians.

### Criteria for discontinuing or modifying allocated interventions {11b}

Chemotherapy dosage modifications will be based on nadir counts and interim non-haematologic toxicities of the preceding cycle, as shown in Supplement 2. Study withdrawal will be allowed upon participants’ request or if the grade 4-5 treatment-related toxicity are found to be ≥20% on an interim analysis.

### Strategies to improve adherence to interventions {11c}

Investigators will perform according to the activity checklist in the patient assessment schedule (Table 1).

### Relevant concomitant care permitted or prohibited during the trial {11d}

Vaccination for coronavirus 2 SARS-CoV-2 (COVID-19) is permitted during the COVID-19 pandemics. Alternative medicines such as herbal treatment or antioxidative agents are prohibited during the trial.

### Provisions for post-trial care {30}

All participants will be followed as routine practice for ancillary and post-trial period. Appropriate and immediate treatment will be given if necessary.

### Outcomes {12}

The primary endpoint is PFS, defined as the period from the date of radiation start to the date of any recurrence, death, or last follow-up. The secondary endpoints include OS which is defined as the interval from radiation start to death due to any cause or last follow-up, LRFFS, DMFS, and treatment-related toxicities. Other prespecified endpoints are surgical outcomes (pathological tumor response, hospital stay, perioperative complications, and mortality) for resectable cases, health-related quality of life, and cost-utility analysis.

Analyses will be conducted on an intention-to-treat and per-protocol basis. The Kaplan-Meier method will be used for survival analysis. Log-rank tests will be used to analyze intergroup differences, and significant factors will be further tested using the Cox proportional hazards regression model to identify independent prognostic factors. Statistical analyses will be conducted using SPSS 22.0 software (IBM, Armonk, NY, USA) and STATA version 11 (STATA Corp., College Station, TX, USA). *P* values of <0.05 will be considered statistically significant.

### Participant timeline {13}

Time schedule of enrolment, interventions, assessments, and visits for participants are shown in Table 2.

**Table 2:**
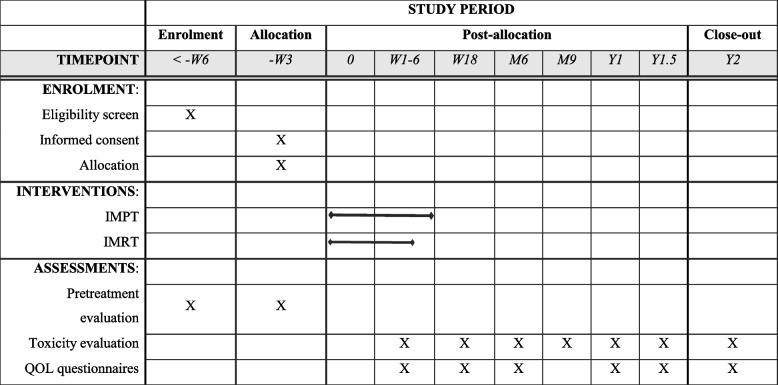
Participant timeline

*IMPT* intensity-modulated proton therapy, *IMRT* intensity-modulated radiotherapy, *QOL* quality of life

### Sample size {14}

The calculated sample size will be 232 patients (116 in each group) to detect a 20% increase in PFS in the high-dose IMPT arm with *α* = 0.05 and power = 80%, corrected for a 10% dropout rate.

### Recruitment {15}

This prospective, multi-institutional nationwide study is performed in 9 academic- and community-based hospitals in Thailand.

## Assignment of interventions: allocation

### Sequence generation {16a}

The allocation sequence will be followed by computer-generated random numbers using block-of-four method. Before randomization, the patients will be stratified according to their resectability status (resectable vs. borderline resectable/unresectable) determined by surgeons.

### Concealment mechanism {16b}

After the screening and recruitment process, the allocation sequence will be implemented by sealed envelopes that are sent from the central institution to the local institutions remotely.

### Implementation {16c}

The stratified allocation sequence will be generated by the project statistician. The research assistant will identify the intervention group in sealed envelopes which are sent to local institutions. Then, the treating physician will assign the intervention accordingly.

## Assignment of interventions: blinding

### Who will be blinded {17a}

During the treatment process, participants and care providers are not blinded because the proton therapy machine is located in a separate building which is clearly named. Treating physicians will be responsible for daily image-guidance IMPT or weekly image-guidance IMRT and will assess toxicity outcomes during treatment on a routine weekly basis. Therefore, the outcome assessors will know the intervention groups. However, the data analysts will be blinded during the statistical analysis.

### Procedure for unblinding if needed {17b}

Not applicable. Only the data analysts will be blinded.

## Data collection and management

### Plans for assessment and collection of outcomes {18a}

Data will be recorded by treating physicians and collected by the research assistance. The central case record forms and questionnaires will be provided in a paper-based and web-based fashion. The standard criteria will be applied, i.e., Common Toxicity Criteria of Adverse Events (CTCAE) version 5.0 for toxicity evaluation and Response Evaluation Criteria in Solid Tumours (RECIST) criteria version 1.1 for tumor response evaluation.

### Plans to promote participant retention and complete follow-up {18b}

After completion of the protocol (2-year follow-up), the participants will continue follow-up annually as routine clinical practice. The treatment outcomes (disease control and toxicity outcomes) will still be collected for participants who complete or discontinue from intervention protocols.

### Data management {19}

All participants will be coded according to their institutional number followed by their sequence. Local institutions will be allowed to use paper-based or web-based case record forms at their convenience. If the paper-based version is used, the research assistants will fill out the electronic case record form.

### Confidentiality {27}

No personal identifiable information will be recorded. Local institutions will assign the participants’ personal code which will be shared with the central institution.

### Plans for collection, laboratory evaluation, and storage of biological specimens for genetic or molecular analysis in this trial/future use {33}

Not applicable. No biological specimens will be collected or kept for genetic or molecular analysis in this trial.

## Statistical methods

### Statistical methods for primary and secondary outcomes {20a}

PFS is defined as the period from the date of RT start to the date of any recurrence, death, or last follow-up. Other secondary endpoints are defined from the date RT start to specific events. Analyses will be conducted on an intention-to-treat and per-protocol basis. The Kaplan-Meier method will be used for survival analysis. Log-rank tests will be used to analyze intergroup differences, and significant factors will be further tested using the Cox proportional hazards regression model to identify independent prognostic factors. Statistical analyses will be conducted using SPSS 22.0 software (IBM, Armonk, NY, USA) and STATA version 11 (STATA Corp., College Station, TX, USA). *P* values of <0.05 will be considered statistically significant.

### Interim analyses {21b}

An interim analysis is planned to be performed when 50% of participants (116 patients) complete a 2-year follow-up. Termination of the study will be decided when grade 4 or 5 toxicity occurs in ≥20% of patients according to the results from the ARTDECO study [11].

### Methods for additional analyses (e.g., subgroup analyses) {20b}

Subgroup analyses will be performed in resectable vs. borderline resectable/unresectable cases.

### Methods in analysis to handle protocol non-adherence and any statistical methods to handle missing data {20c}

Missing data will be handled by multiple imputation method.

### Plans to give access to the full protocol, participant-level data, and statistical code {31c}

The participant-level dataset and statistical code will be available upon request.

## Oversight and monitoring

### Composition of the coordinating center and trial steering committee {5d}

Before the trial starts, the trial steering committee, composed of radiation oncologists and medical physicists from all 9 institutions, has a meeting to set up the protocol and a workshop to practice delineation, treatment planning, and plan evaluation. Then, a regular 6-monthly meeting will be scheduled.

### Composition of the data monitoring committee, its role, and reporting structure {21a}

Data monitoring committee (DMC) composes of radiation oncologists and a biostatistician to perform an interim analysis. We declare that DMC are independent from the sponsor and had no competing interests.

### Adverse event reporting and harms {22}

Toxicities will be assessed weekly during CRT and adjuvant chemotherapy every 3 months × 3 visits after treatment completion, every 6 months × 2 visits, and then annually using version 5 (CTCAE). Grade 4–5 toxicity will be centrally reported and the study will be terminated when grade 4–5 toxicity occurs in ≥20%.

### Frequency and plans for auditing trial conduct {23}

Project Management Group has a monthly meeting to review trial conduct. The Trial Steering Group and the independent Data Monitoring and Ethics Committee have a meeting every 6 months to review conduct throughout the trial period.

### Plans for communicating important protocol amendments to relevant parties (e.g., trial participants, ethical committees) {25}

According to the institutional review board (IRB) regulations, the protocol must be reviewed and renewed annually.

### Dissemination plans {31a}

The results of the study will be published in an international journal.

## Discussion

Dosimetric and clinical benefit of PBT in comparison with IMRT in the treatment of esophageal cancer has been demonstrated in several retrospective studies [17, 18] as well as a phase IIB randomized study [22]. However, the results from these studies are still limited to adenocarcinoma of the lower esophagus. There exist differences in histology, location, dose-response relationship, and potential for resectability between adenocarcinoma and squamous cell histology. Furthermore, the highly conformal technique, IMPT, using high-dose radiation has yet been studied in any prospective study.

Therefore, this multi-institutional phase II/III RCT aims to compare overall treatment outcomes between high-dose IMPT and standard-dose IMRT for the treatment of esophageal cancer. We expect that dose escalation (60–64Gy) can be administered safely using modern technique proton therapy and may improve tumor control and survival outcomes in an endemic area of squamous cell carcinoma. The final goal of this study is to establish high-level evidence for the implication of IMPT for ESCC treatment.

## Trial status

Protocol version 1, date 10 March 2020.

Date of recruitment began: 1 August 2021

Approximate date of recruitment complete: 31 July 2026

## Supplementary Information

**Additional file 1: Supplement 1.** Target volumes and normal structure delineation and constraints. **Supplement 2.** Chemotherapy dosage modifications protocol.

## Data Availability

The datasets used and/or analyzed during the current study are available from the corresponding author on reasonable request.
