# Supplementary material for: High-dose Intensity-modulated proton therapy versus Standard-dose Intensity-modulated RadIation therapy for esophageal squamous cell carcinoma (HI-SIRI): study protocol for a randomized controlled clinical trial
Source: Trials. 2022 Oct 22;23:897. doi: 10.1186/s13063-022-06822-8 (PMC9587557; doi:10.1186/s13063-022-06822-8)
Supplement: Supplementary file 1 — Additional file 1: Supplement 1. Target volumes and normal structure delineation and constraints. Supplement 2. Chemotherapy dosage modifications protocol. [file 13063_2022_6822_MOESM1_ESM.docx]

**Supplement materials**

**Supplement 1. Target volumes and normal structure delineation and constraints**

Gross target volumes (GTV) consist of gross primary tumor (GTV-P) with the whole circumferential wall of the involved esophagus, and pathologic nodes (GTV-N). High-risk clinical target volume (CTV-HR), or boost volume, includes the GTV-P with a 0.5-cm radial and a 2-cm craniocaudal margin along the esophagus, and the GTV-N with a 0.5-cm circumferential margin. Low-risk CTV (CTV-LR) encompasses the GTV-P with a 0.5-cm radial and a 4-cm craniocaudal margin (for lower esophageal cancer, distal margin is restricted to 2 cm extending into gastric cardia), the GTV-N with a 0.5-cm circumferential margin, and elective nodal region. For the proximal third esophagus, the paraesophageal lymph nodes (LNs), bilateral paratracheal LNs, and bilateral supraclavicular LNs are treated. For middle lesions, the paraesophageal LNs, bilateral paratracheal LNs, and subcarinal LN are treated. For the lesion at distal esophagus and gastroesophageal junction, the paraesophageal LNs, lesser curvature LNs, gastrohepatic and celiac LNs are treated. For the supraclavicular lymph node metastasis, treatment of higher echelon cervical nodes are considered.

For IMPT plan, robust optimization is applied to the CTVs to account for uncertainties and interplay effect with 3.5% calibration curve error and 5 mm isocenter shift. For IMRT plan, planning target volumes (PTVs) is created from CTVs plus a 1-cm isotropic margin to account for setup error. All PTVs is at least 0.7 cm away from the spinal cord and 0.5 cm from the skin. Lungs, heart, liver, and spinal cord are contoured for all patients, but kidneys are delineated for those with CTVs beyond gastroesophageal junction.

Dose constraints are summarized in the table.

| **Structures** | **Dosimetric parameters** | **Per protocol** | **Variation acceptable** |
| --- | --- | --- | --- |
| **CTVs (for IMPT)** | D99% | 100% | - |
| **PTV HR (for IMRT)** | V100% | 95% | 90% |
|  | D99% | ≥93% | ≥90% |
|  | D0.03cc | ≤110% | ≤115% |
| **PTV LR (for IMRT)** | V100% | ≥95% | ≥90% |
| **Spinal cord** | Dmax | ≤45 Gy | ≤ 50 Gy |
| **Lungs – GTV** | V20 Gy | <35% | <40% |
|  | V10 Gy | <40% | <60% |
|  | Mean dose | <20 Gy | - |
| **Heart** | V30 Gy | <30% | <40% |
|  | Mean dose | <30 Gy | - |
| **Liver** | V30 Gy | <30% | - |
|  | Mean dose | <25 Gy | - |
| **Kidneys** | D33% | <18 Gy | - |
| **Stomach and small bowel** | Mean dose | <30 Gy | - |
|  | Dmax | <maximal PTV dose |  |

*IMPT=intensity modulated proton therapy; IMRT=intensity modulated radiotherapy; GTV=gross target volume; CTV=clinical target volume; PTV=planning target volume; HR=high-risk; LR=low-risk; Dmax = maximum dose; Vx= volume receiving x Gy (RBE)*

**Supplement 2. Chemotherapy dosage modifications protocol**

| **Toxicity** | **Grade** | **Agent** | **Modification** |
| --- | --- | --- | --- |
| ANC nadir < 500 for ≥ 5 days and/or platelet nadir < 50,000 | Grade 4 ANC and/or  Grade 3 platelets | 5-FU/cisplatin  Paclitaxel/carboplatin | Decrease 20% |
| ANC < 1,000 or platelet < 50,000 | Grade 3 ANC and/or  Grade 3 platelets | RT/Chemo | Hold for 1 week |
| Infection or bleeding related to  myelosuppression | Present | 5-FU/ cisplatin  Paclitaxel/carboplatin | Decrease 20% |
| Serum creatinine | > 1.4 but < 2.0 | cisplatin | Decrease 50% |
| Serum creatinine | ≥ 2.0 | cisplatin | Discontinue |
| Neurotoxicity | Grade ≥3 | cisplatin | Discontinue |
| Ototoxicity | Grade ≥3 | cisplatin | Discontinue |
| Fatigue | Grade 4 (≥ 5 day) | cisplatin | Discontinue |
| Mucositis/esophagitis | Grade 4 | RT/Chemo | Hold for 1 week |
| Vomiting/diarrhea/  dehydration | Grade 4 | cisplatin | Decrease 20% |
| Any non-hematological or other toxicity/adverse event, except alopecia, nausea/vomiting | Grade ≥3 | 5-FU | Hold on treatment week and resume at 240 mg/m^2^/d Monday-Friday (96 hours) for the remaining duration of therapy (providing  the toxicity has resolved to grade ≤1) |

*ANC=absolute neutrophil count; RT=radiotherapy; 5-FU=5-fluorouracil*
